# Discrete Element Method Analysis of the Spreading Mechanism and Its Influence on Powder Bed Characteristics in Additive Manufacturing

**DOI:** 10.3390/mi12040392

**Published:** 2021-04-02

**Authors:** Valerio Lampitella, Marco Trofa, Antonello Astarita, Gaetano D’Avino

**Affiliations:** Dipartimento di Ingegneria Chimica, dei Materiali e della Produzione Industriale, Università degli Studi di Napoli Federico II, Piazza Giorgio Ascarelli 80, 80125 Napoli, Italy; marco.trofa@unina.it (M.T.); antonello.astarita@unina.it (A.A.); gaetano.davino@unina.it (G.D.)

**Keywords:** discrete element method, spreading process, additive manufacturing

## Abstract

Laser powder bed fusion additive manufacturing is among the most used industrial processes, allowing for the production of customizable and geometrically complex parts at relatively low cost. Although different aspects of the powder spreading process have been investigated, questions remain on the process repeatability on the actual beam–powder bed interaction. Given the influence of the formed bed on the quality of the final part, understanding the spreading mechanism is crucial for process optimization. In this work, a Discrete Element Method (DEM) model of the spreading process is adopted to investigate the spreading process and underline the physical phenomena occurring. With parameters validated through ad hoc experiments, two spreading velocities, accounting for two different flow regimes, are simulated. The powder distribution in both the accumulation and deposition zone is investigated. Attention is placed on how density, effective layer thickness, and particle size distribution vary throughout the powder bed. The physical mechanism leading to the observed characteristics is discussed, effectively defining the window for the process parameters.

## 1. Introduction

Additive Manufacturing (AM) has become a widespread method for the production of parts in fields such as aerospace and biotechnology where both a complex shape and good mechanical properties are required [1]. The term ’Additive Manufacturing’ refers to a large family of technologies. The most popular ones in industrial applications are the powder bed-based techniques, where a thin layer of particles, ranging from few microns to several dozen, is spread by a re-coater and successively sintered or melted by either a laser or an electron beam; the process is then repeated until the part is fully built [2]. Given the strong industrial interest for this technology, a great number of studies has been carried out to highlight and understand the underlying physical mechanism. Due to the complex multiphysics nature of the process, the effect of the manufacturing parameters on the mechanical properties of the final part is not completely understood. Indeed, some unsolved issues remain such as process repeatability, internal defects of the printed parts, and non-uniformity of the properties within the building chamber [3].

The research activity on AM is mainly focused on the melting/sintering process, neglecting the spreading of the powders to build the powder bed. The most commonly considered features are the scan strategy, the characteristics of the powders, and the parameters of the heat source [4]. However, the characteristics of the powder bed (e.g., the packing factor, its homogeneity within the powder layer, and the actual layer thickness) strongly influence the printing process and must be carefully taken into account. More specifically, the spreading process remains the only form of control on the state of the powder bed before the sintering step. An experimental approach can be quite challenging due to the lack of standardized procedures to evaluate the quality of the powder bed. Therefore, the research activity has mainly relied on numerical simulations to solve the longstanding problem of the powder bed deposition.

In this regard, the Discrete Element Method (DEM), first proposed by Cundall and Strack [5], has been widely used in the simulation of granular systems [6,7] and suspensions [8,9] and has been proven to be suitable to accurately predict the powder spreading in Laser Powder Bed Fusion (LPBF) techniques. Parteli and Paschel [10] developed a numerical tool that explicitly takes into account the complex shape of the particles and used it to investigate the effect of a varying coating speed and particle size distribution on the surface roughness. Moreover, the load on the partially built part was also monitored. Mindt et al. [11] focused on the spreading of commercial Ti-6Al-4V powder, comparing the results with that of simpler `rain’ models. The effect of the coating speed and building plate displacement on the powder packing density was investigated. In the work from Haeri et al. [12], the focus was on how different aspect ratios of the particles influence the roughness and the solid volume fraction of the powder bed, showing that optimal results can be achieved for an aspect ratio of 1.5. It was also shown that a roller performs better than a blade in terms of geometry of the spreading device. These findings are qualitatively confirmed by experiments, even though the experimental setup does not realistically reproduce the spreading conditions. Chen et al. [13] developed a numerical model to investigate the powder flowing behavior, explicitly taking into account the cohesive forces between the particles. Decreasing the particle size was beneficial for the fluidity of the powder bed. However, for particles with radius lower than 21.8 μm, the effect of the cohesion forces becomes prominent, hindering the powder flowability. An experimental validation was carried out, comparing the simulated and the real profile of the powder heap in front of the blade during the spreading process. Similar findings by Meier et al. [14] confirmed that if the median diameter of the particles is too small, the resulting powder bed quality can be compromised. Haeri [15] used a DEM model to identify more efficient shapes for the spreading device that can lead to a quasi-critical value of powder bed density. Nan et al. [16] studied the jamming caused by the highly frictional nature of the powders used in AM. The frequency and duration of the jamming phenomena were related to the layer thickness. Zhang et al. [17] employed a DEM model to simulate ceramic powder roller-spreading with specific attention to the influence of the layer thickness and of the roller parameters on the packing factor of the powder bed. Chen et al. [18] focused on the deposition mechanism at a particulate scale and tried to establish a comprehensive model. Three competing mechanisms which can influence the packing density of the powder bed were identified: the cohesion effect, the wall effect, and the percolation effect. A similar approach can be found in Fouda et al. [19], showing that the final deposited layer packing fraction is influenced by the dilation caused by the starting of the spreader, the rearrangement due to the crossing of the gap, and the inertia of the already deposited particles. Furthermore, the relationship between the process parameters and the relative influence of those mechanisms was also highlighted. Han et al. [20] tried to establish a systematic approach for the optimization of the layer thickness. A numerical model was developed to analyze the effect of the layer thickness on the powder bed density and the optimum parameters were used to produce samples whose microstructure and tensile strength were assessed. Desai et al. [21] employed an interesting approach to overcome the limitation of the high computational power required by DEM method by using the DEM-based simulations to train a feed forward, back propagation neural network which was then used to study the relationship between spreading parameters and process results.

It is worthwhile to point out that the widely used discrete element method has several parameters to be fixed. In most of the aforementioned studies, a calibration procedure to select these parameters is missing. Consequently, while the results are still useful to obtain a qualitative insight on the process, the developed models are not reliable for predictive purposes. Another common misconception is the use of a single process parameter (e.g., the dynamic angle of the powder heap in front of the blade) to both calibrate and validate the model, thus affecting the thoroughness of the model itself. Finally, most of the works on the topic only focuses on the deposition stage of the process, starting from a non-realistic configuration, such as a boulder of powder generated in front of the blade, or simply leveling a thick layer of powder.

In the present work, we build up and run a DEM model for simulating the powder spreading process with the aim of analyzing in detail the motion of the powder bed from the often-neglected accumulation stage (where the powder heap is built) to the deposition stage, investigating the effect of the spreading process on the state of the powder reservoir. An accurate estimate of the DEM model parameters is carried out through a calibration stage with independent experiments. The spreading process is simulated considering the effective layer thickness. In this regard, most of the literature results are referred to layers that are of the same dimension of the mean diameter of the powders. However, due to phenomena like powder compaction and thermal contraction of the already printed part, it can be significantly larger [22]. The main spreading mechanism is studied for two velocities of the re-coating device corresponding to two different flow regimes. The resulting powder bed characteristics (i.e., density, effective layer thickness, and mean diameter of the powder) and their variation along the spreading direction is presented and discussed, highlighting the physical mechanism leading to the observed features of the deposited powder.

## 2. Materials and Methods

### 2.1. Discrete Element Method

The discrete element method is a technique able to simulate the time evolution of a system made by a relatively large number of particles in contact. It consists of computing the forces acting on each particle due to the contacting particles and external forces (in our case only gravity). At each time step, the translational and angular velocity of the *i*-th particle, ${\vec{u}}_{i}$ and ${\vec{\omega }}_{i}$, are calculated from the force and torque balances:

(1) $$m_{i}\frac{d{\vec{u}}_{i}}{dt}=\sum_{j}{\vec{F}}_{ij}+m_{i}\vec{g}\;\;I_{i}\frac{d{\vec{\omega }}_{i}}{dt}=\sum_{j}{\vec{T}}_{ij}$$

where $m_{i}$ and $I_{i}$ are the particle mass and moment of inertia, $F_{ij}$ and $T_{ij}$ are the interaction force and torque on the *i*-th particle due to the other *j*-th particles or the wall, and $\vec{g}$ is the gravity vector. The particle position $x_{i}$ and rotation angle $\theta_{i}$ are then updated by integrating the following kinematic equations:

(2) $$\frac{d{\vec{x}}_{i}}{dt}={\vec{u}}_{i}\;\;\frac{d{\vec{\theta }}_{i}}{dt}={\vec{\omega }}_{i}$$

Two particles are considered in contact when the distance between their centers is less than the sum of their radii, the difference between these two quantities defining the normal overlap $\delta_{n}=R_{i}+R_{j}-∥{\vec{x}}_{i}-{\vec{x}}_{j}∥$. The relative velocity between the contacting particle surfaces allows to define the unit tangential vector $\vec{t}$ and the tangential overlap $\delta_{t}$ [23].

The contact force is computed through the Hertz–Mindlin model [24], modified to take into account the cohesion of the particles [6,9]. Cohesion forces are indeed relevant for particles smaller than about 100 μm as they are comparable with the gravity force [25]. The normal and tangential components of the contact force are:

(3) $$F_{ij,n}=k_{n}\delta_{n}+\gamma_{n}\frac{d\delta_{n}}{dt}-4\pi kR_{r}\delta_{n}$$

(4) $$F_{ij,t}=-\left(k_{t}\delta_{t}+\gamma_{t}\frac{d\delta_{t}}{dt}\right)$$

where *k* is the cohesion energy density. The tangential force is limited by the Coulomb criterion $\left|F_{ij,t}\right|\leq \mu_{s}\left|F_{ij,n}\right|$ with $\mu_{s}$ the sliding friction coefficient. The other parameters in Equations (3) and (4) are linked to the material properties trough the following expressions:

(5) $$k_{n}=\frac{4}{3}E_{r}\sqrt{R_{r}\delta_{n}}$$

(6) $$k_{t}=8G_{r}\sqrt{R_{r}\delta_{n}}$$

(7) $$\gamma_{n}=-2\frac{\ln\epsilon }{\sqrt{\ln^{2}\epsilon +\pi^{2}}}\sqrt{\frac{5}{3}m_{r}E_{r}\sqrt{R_{r}\delta_{n}}}$$

(8) $$\gamma_{t}=-4\frac{\ln\epsilon }{\sqrt{\ln^{2}\epsilon +\pi^{2}}}\sqrt{\frac{5}{3}m_{r}G_{r}\sqrt{R_{r}\delta_{n}}}$$

where $m_{r}$, $R_{r}$, $E_{r}$, and $G_{r}$ are the equivalent mass, radius, Young’s modulus, and shear modulus, and ϵ is the coefficient of restitution. The coefficient of restitution is defined as the ratio between the kinetic energy of two objects before and after a collision and therefore denotes how fast the energy of the system is dissipated during the impacts between particles.

The torque in Equation (1) contains a sliding component $R_{i}F_{ij,t}$ and a rolling contribution $\mu_{r}R_{r}k_{n}\delta_{n}$ (constant directional torque model [26]) where $\mu_{r}$ is the coefficient of rolling friction.

To guarantee numerical stability the time step is chosen less than 15% of the Rayleigh time [23]. The simulation is performed by the open source software LIGGGHTS 3.8.0 [27].

### 2.2. Simulation Setup

**Figure 1:**
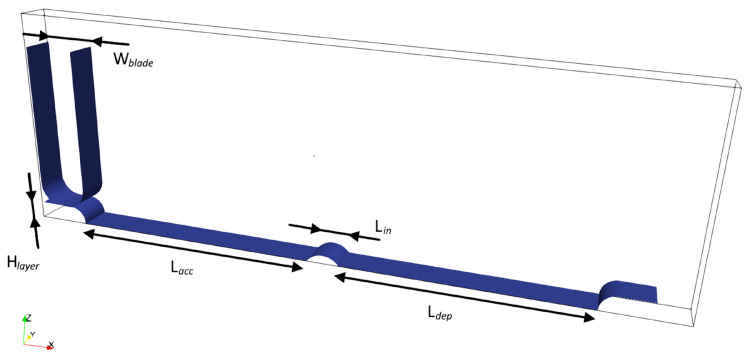
Setup used for the simulations with relevant dimensions.

The computational domain, shown in Figure 1, faithfully reproduces the powder spreading device of an actual 3D printer. Two distinct zones can be identified: the accumulation zone, where the powder particles are collected and accumulate to form a growing heap in front of the blade, and the deposition zone, where the heap is partially stratified in a thin layer. A qualitative analysis on the mechanisms involved in the powder heap accretion will be carried out to identify the arising of segregation processes or any other phenomenon that could be detrimental for the powder bed quality. Ideally, the thickness of the deposited layer is equal to the gap between the blade and the building plane (i.e., the one where the powder is deposited). Actually, it can be up to twice larger, due to powder compaction and thermal contraction of the already printed part [11,22]. For these reasons, in the simulation the gap was set to 100 μm, larger than the one usually adopted for a real printing process.

We assume periodicity in the direction orthogonal to the movement of the blade (*y*-direction in Figure 1). Therefore, only a narrow slice of the whole device is simulated, assigning a periodic boundary condition to the lateral boundaries (corresponding to the planes of the computational box parallel to the xz-plane). We have verified that the periodic condition does not alter the results by progressively increasing the domain width and comparing the average results. The building plane is considered perfectly flat, while all the edges have been rounded to improve the stability of the simulation. The dimensions of the setup are listed in Table 1. Even though the dimensions are fairly smaller compared to that of an actual 3D printing system the length was sufficient to reach a steady state spreading conditions (see later) and therefore was used in the simulations.

### 2.3. Material Properties

To run simulations of a complex phenomenon (such as the powder spreading in AM) in a reasonable time, some approximations are required [28]. The simplifications can apply to both the material properties (e.g., with a reduction of the particle stiffness [29]) and the geometry of the simulation (e.g., by exploiting periodicity or simplifying the particle shape). Of course, the material properties need to be properly calibrated to reproduce the real behavior of the powder and capture the essential physical mechanism of the spreading process.

The material for the powders considered in this study is Inconel718, a Nickel-based superalloy that is largely used in AM applications [30]. Given the good degree of roundness of the powders, in the simulations the particles will be considered perfectly spherical, thus avoiding the added complexity of a multi-sphere model. This approximation holds true for virgin water-atomized powders [31].

Since the particle size distribution has a relevant influence on the spreading process behavior [32], we have reproduced the distribution of a powder typically used for AM. Specifically, the experimental particle size distribution has been discretized by considering particles with four different sizes, i.e., 13, 21, 28, and 42 μm, with number fraction 0.2, 0.2, 0.5, and 0.1, respectively, resulting in an average diameter of $D_{m}\approx 25$ μm. Notice that the distribution used in the simulation results from the truncation of the smallest particles, as their inclusion would have required a much smaller time step to preserve numerical stability.

As previously discussed, several material parameters need to be specified in a DEM simulation. Some of them are taken from the literature and are listed in Table 2. Notice that as compared to the real value, the Young’s modulus has been reduced by several orders of magnitude, allowing the use of a larger time step (Δ*t* = 4 × 10^−8^ s) and a reduction of the computational cost, without altering the overall behavior of the powder spreading mechanism [29].

The other two relevant parameters, i.e., the coefficient of sliding friction $\mu_{s}$ and the cohesion energy density *k*, will be estimated through the calibration procedure discussed below.

## 3. Calibration

The problem of DEM calibration has been extensively discussed in the literature and various solutions have been proposed [25,28,33,34]. One of the major issues when calibrating a DEM model is that the calibration itself can absorb a quantity of computational resources comparable with the main simulation [35]. Therefore, the setup used for calibration should be as simple as possible.

To avoid excessive complications, in this work we will consider two target values for the comparison between experiments and simulations, namely the angle of repose (i.e., the steepest stable angle formed by a pile of powder) and the angle of slipping (i.e., the angle at which a fine layer of powder slips from a flat surface). Both these quantities have been used in the literature to characterize the flowability of bulk materials [33].

To maintain the correspondence between the number of target values and the calibrated quantities we will consider two varying parameters: the sliding friction coefficient $\mu_{s}$ and the cohesion energy density *k*. The coefficient of rolling friction is set to such a value that the rolling of a particle stops after a distance of approximately 15–20 times its radius [36]. Since the rolling friction is mostly used to account for the non-spherical shape of the powder and for the mechanical interlocking that can arise, a fine tuning of this parameter was deemed not necessary.

The powder was kept in a sealed container to control humidity and dried before the calibration. The angle of repose $\theta_{\mathrm{rep}}$ was measured by manually pouring the powder on a round-shaped base of known diameter *d* until it reached a maximum height *h*. The height of the pile was then measured, and the angle of repose was obtained as:

(9) $$\theta_{\mathrm{rep}}=\arctan\left(\frac{h}{d/2}\right)$$

The test was repeated three times and the mean value obtained for the angle of repose was about 28.7°.

Another simple experimental test was carried out to obtain the slipping angle for the selected powder: a thin layer of powder was spread on a flat surface which was slowly tilted until the powder started slipping uniformly. At this point, the tilting was stopped, and the angle recorded. Again, the test was repeated three times and the value obtained for the slipping angle was about 41.6°.

The aforementioned angles have been used as target values for the calibration of the DEM parameters. The simulation setup for the calibration tests faithfully reproduces the experimental one. However, to reduce the overall computational time needed to perform the calibration tests, we adopt the `cloud method’ [37] for the determination of the angle of repose: the particles are not poured through a funnel, but are generated in a loose cloud above the base and let settle until a stable angle is attained. Despite the simplification, this method has shown a good accuracy and a considerable saving of computational time.

**Figure 2:**
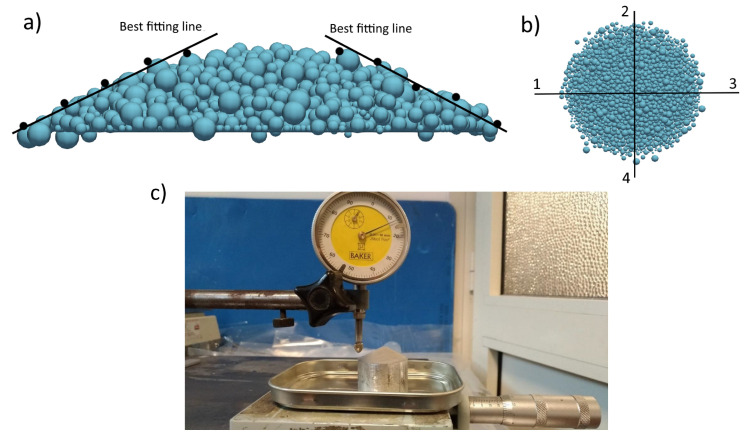
Procedure for the determination of the angle of repose of the powder pile. (**a**) Example of how the best fitting line is obtained (**b**) The orthogonal directions along which the angle of repose is computed. (**c**) Experimental setup for the calculation of the angle of repose.

While the slipping angle can be straightforwardly obtained, the calculation of the angle of repose required a post-processing strategy, since the relatively small number of powder particles in the pile leads to a rougher surface compared to the real one. As shown in Figure 2a, we select five equally spaced points belonging to the powder surface. The angle between the line that fits these points, and the horizontal plane is the angle of repose. The process is repeated for each pile along the directions shown in Figure 2b and the average of the four values is used as angle of repose. The experimental setup for the calculation of the angle of repose is presented in Figure 2c.

**Figure 3:**
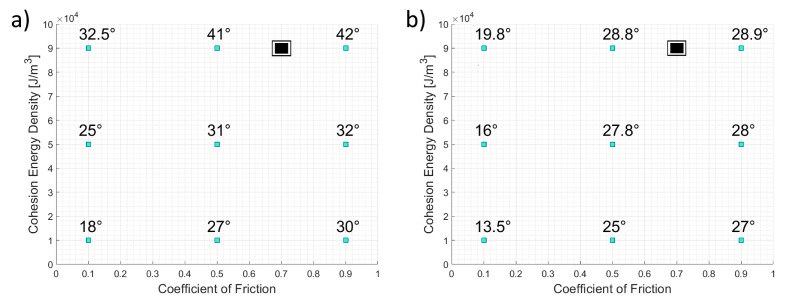
Effect of the variation of the coefficient of friction and cohesion energy density on the slipping angle (**a**) and angle of repose (**b**). The black rectangle is the best combination that reproduces the target values.

We run simulations with nine combinations of coefficient of friction and cohesion energy density, as reported in Figure 3, to link them to slipping angle and angle of repose. The bounds of the parameter space were set based on the existing literature [25,28,33,34] and on the observation of non-physical behavior for values beyond these limits. The experimental target values are attained for the calibration parameters falling in the upper right portion of the figures. We run other simulations in this region to refine the estimate, finding $\mu_{s}=0.7$ and $k=90,000\;J/m^{3}$ (denoted by the black rectangle Figure 3) with a relative error around 4% for both quantities.

## 4. Results

The next sections will focus on the phenomena occurring during the accumulation and deposition phases of the spreading process and how such phenomena influence the state of the powder reservoir and the characteristics of the powder bed. Moreover, the influence of the speed of the re-coating device on the spreading mechanism will be studied. In this regard, the velocity of the blade is set to two values to investigate two different regimes of motion. Specifically, we set the lowest value to 20 mm/s, corresponding to a quasi-static regime, and the highest value to 100 mm/s, where inertial effects play a significant role [14].

The other parameters are kept fixed since the main scope of the present work is to identify the underlying mechanism of the powder spreading process and not to perform a parameter optimization. For the powder reservoir, the total mass of particles remaining after the spreading process will be considered, alongside with the variation of the mean diameter of the particles. In addition to these parameters, for the deposited powder bed, the effective layer thickness and the local density will also be considered and their variation throughout the powder bed will be investigated.

To avoid any possible influence of the initial state of the powder bed on the results, every simulation is repeated with three different starting conditions, obtained by varying the initial position of the particles. Therefore, the results will be plotted taking into consideration the mean values as well as the standard deviations (reported as error bars). Each iteration, Including the insertion, the settlement of the powder in the reservoir and the actual spreading required slightly less than 24 h of computational time.

### 4.1. Accumulation Stage

**Figure 4:**
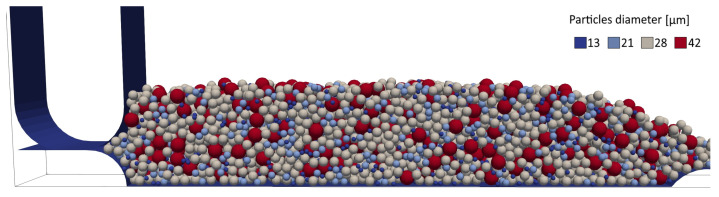
State of the powder feedstock after the settling of the particles.

To generate the feedstock of powders for the spreading process, 4500 particles are generated above the accumulation zone and are let settle under the action of gravity until they stop moving. The quantity of powder for the feedstock is chosen to guarantee that the powder entirely covers the deposition zone at the end of the spreading process. An example of the initial powder feedstock is displayed in Figure 4.

As previously mentioned, in the accumulation zone, the blade collects the deposited powder and the heap in front of the blade grows. As the blade starts to move, it comes into contact with the mass of powder in the reservoir which is usually thicker than the layer to be deposited to ensure the completion of the process. In the first instants of the accumulation process, the particles move upward along the blade causing a sharp rise on the height of the powder heap.

**Figure 5:**
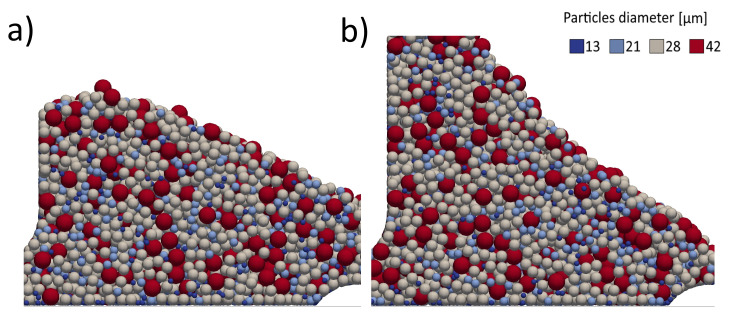
Dynamic angle of repose in the accumulation stage for two different speeds of the blade: (**a**) 20 mm/s, (**b**) 100 mm/s.

After a brief transient, the process achieves a stationary state with the powder reaching a characteristic dynamic angle of repose. The value of this angle appears to be determined by the concurrent action of two contrasting phenomena: the obstruction caused by the particles in front of the powder heap that promotes a steeper angle and the effect of gravity that causes the rolling and rearrangement of the particles in a more acute avalanche. Since the resistance of the particles in front of the blade is more relevant at higher speeds, the dynamic angle of repose increases with the velocity of the blade from around 25° to over 36° as shown in Figure 5, where the typical angle at which the particles arrange can be observed. Once the stationary state is attained, as more particles are swept, the powder heap grows without any alteration of the shape. In this state, the mechanism of the powder heap accretion can be identified.

Focusing the attention on the zone in front of the re-coating device, it can be noted that during this stage, the trajectory of the particles varies significantly based on their diameter. While the bigger particles tend to get caught in the powder heap, the smaller ones can fill in the gaps left behind and, therefore, sink into the powder reservoir with a mechanism somewhat similar to that observed in granular convection [38]. This phenomenon shows the existence of vertical interactions able to influence the state of the powder reservoir and of the successive layers. Indeed, after the spreading process, the mean diameter of the powders in the reservoir slightly drops from the initial value of ≈25 μm to a value of ≈23 μm.

**Figure 6:**
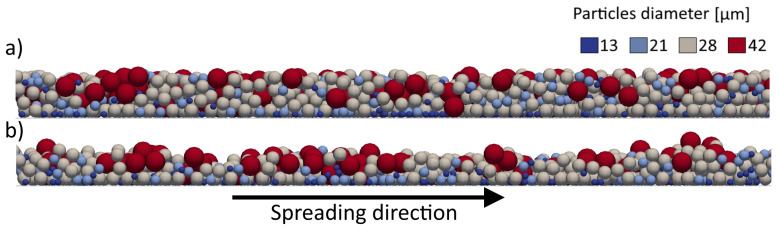
State of the powder reservoir after the spreading for two different speeds of the blade: (**a**) 20 mm/s, (**b**) 100 mm/s.

The speed of the blade has a relevant effect on this phenomenon. A visual analysis of the powder reservoir after the spreading process for different speeds of the blade presented in Figure 6 shows the formation of discontinuities and an uneven distribution of particles. Such discontinuities, while not directly influencing the spreading process of the current layer, can lead to a variation in the total amount of powder that is going to be spread in the next layer and can therefore cause defects such as short feed. Indeed, the mass of powder left in the reservoir after the spreading decreases of about 27% for the highest speed of the blade. Therefore, to guarantee the spreading of the same amount of powder for each layer, a larger amount of powder is involved in a whole printing process, increasing the costs related to the recovery of unmelted particles.

### 4.2. Deposition Stage

**Figure 7:**
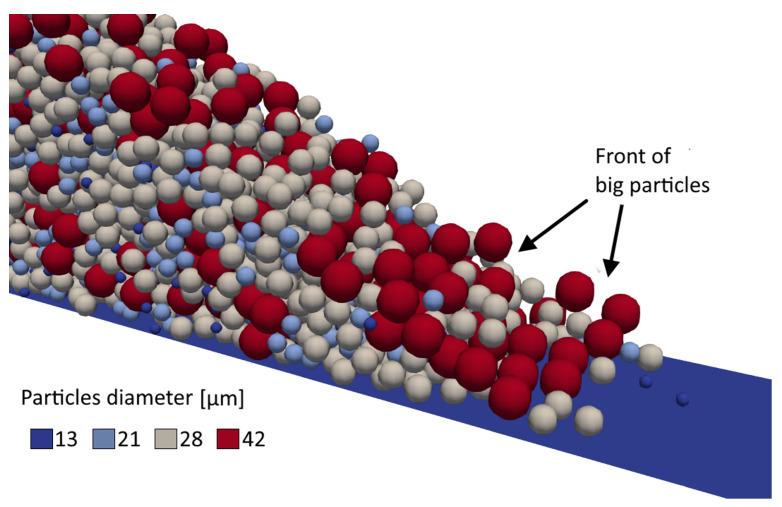
Snapshot of the base of the powder heap, where a front formed by the bigger particles can be observed. The picture is referred to a 20 mm/s spreading speed.

Once the blade reaches the deposition zone, the lack of new particles on the trajectory of the blade leads to an alteration of the heap equilibrium, with a sharp decrease of the dynamic angle of repose. Moreover, the bigger particles located on the free surface of the powder heap, being the most susceptible to the effect of gravity, are dragged down and accumulate at the base of the pile, as displayed in Figure 7. This phenomenon leads to a segregation process whose effects will be found in the final powder bed. The figure clearly shows the accumulation of bigger particles (in red) towards the front of the pile, more evident at lower blade speed due to the relatively higher contribution of gravity as compared to other forces.

As already mentioned, a higher powder bed density is desirable since a lower void fraction leads to better mechanical properties of the printed parts. However, what is more important than the absolute value of the density is its homogeneity, i.e., the variation throughout the entire powder bed. Indeed, the parameters of the laser or the electron beam used to process the powder bed (e.g., hatch spacing and power) must be carefully selected to obtain the right power energy density for a proper melting. In case the powder bed is characterized by zones with significantly different density, defects such as keyhole mode may appear [3]. The same argument applies for the total amount of powder present in the different zones of the powder bed, where an excessive accumulation in certain zones can lead to issues and defect formation during the laser processing stage.

To quantitatively characterize the state of the powder bed after the spreading process, we divide it into 15 equally spaced sub-regions along the spreading direction. In each zone, we calculated the relative density, the effective layer thickness, and the mean diameter along the spreading direction. The effective layer thickness is obtained as a mean along the *z*-axis of the upper border of the particles that lie on the surface of the powder pile. The relative density is defined as the total mass of the particles divided by the volume of the theoretical layer, and normalized with the density of the bulk material. The volume of the theoretical layer is calculated as the volume of the rectangular parallelepiped with the height of the effective layer thickness, the width of the simulation box, and the length of the sub-region. Finally, the mean diameter is the average of the diameters of the particles with center within a sub-region.

**Figure 8:**
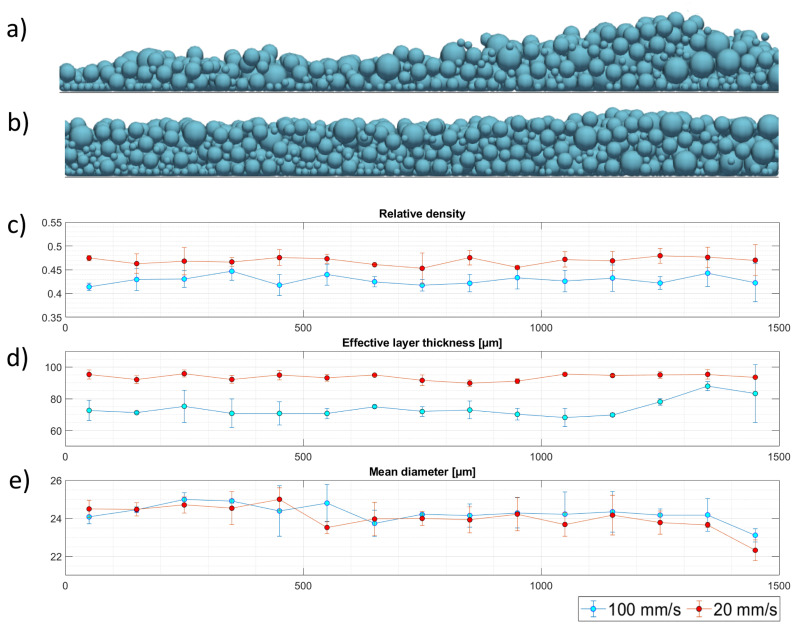
Typical state of the powder bed after the spreading process at (**a**) 100 mm/s and (**b**) 20 mm/s. (**c**) Relative density, (**d**) effective thickness, and (**e**) mean diameter of the layer of powder along the spreading direction.

Figure 8 shows the state of the powder bed (panel a and b) and the three aforementioned quantities along the spreading direction (panels c–e) at the end of the spreading process for the higher and lower blade speed. Since the re-coating device interacts with the particles, it can be assumed that the particles have a velocity in the spreading direction that is a fraction of the speed of the blade itself. As a result, for the high-speed case, the formation of a void can be observed in the first part of the powder bed where the effective layer thickness is much lower than the gap size (100 μm) and, at the same time, the particles tend to accumulate in the ending zone of the powder bed where the effective layer thickness grows significantly. Hence, for high-speed values, the movement of the blade has a strong influence on the formation of such discontinuities (a similar behavior can be observed in Reference [14]). Another critical zone is the end of the powder bed, where the relative density shows an increased variability (bigger error bars). More specifically, this phenomenon can be related to the combination of two factors: the so-called ‘rebound effect’ which was also observed by Han et al. [20] for significantly smaller values of the layer thickness and the aforementioned particle segregation. The rebound effect is caused by the presence of a solid obstacle (i.e., the wall at the end of the deposition zone) in the trajectory of the blade. As the blade approaches this obstacle, the particles caught in between give rise to force arcs. When the movement of the blade causes the breaking of these force arcs, the particles are expelled at relatively high speed and alter the quality of the already deposited powder bed. While the impact of such phenomenon should be negligible because it takes place in a small region at the end of the powder bed that is not used for actual print processes, the results hint that a similar mechanism could somehow come into play any time there is an interaction between the loose powder and a solid object (i.e., the already printed part). A similar argument holds also for ‘voids’ observed at the beginning of the powder bed and, therefore, a more detailed research on the aforementioned interaction is needed, which will be addressed in a future work. Apart from these zones, a slower spreading speed leads to a more stable layer thickness and to a larger relative density, leading to a higher quality of the powder bed. Anyhow, a stronger variability of the powder bed characteristics can be observed in the zone affected by the rebound.

From Figure 8e, it can be seen that the mean diameter of the powder reduces throughout the powder bed, further confirming the arise of segregation phenomena, similarly to what was observed in the accumulation stage. The reduction of the mean diameter in the initial portion of the powder bed observed in Figure 8e can be explained noting that the biggest particles have a bigger momentum when dragged by the blade therefore move farther in the spreading direction and for higher speeds of the blade this appears to be slightly more evident. However, in the rest of the powder bed, at variance with what was observed for the effective layer thickness and the relative density, a lower spreading speed promotes the aforementioned segregation phenomena, thus leading to a more stable trend that results in a variation of the size distribution throughout the powder bed. More specifically, the mean diameter constantly decreases alongside the spreading direction, since the big particles accumulated at the base of the powder heap are deposited first, leaving an overall finer powder for the remaining part. While the phenomenon can overall be observed even at a higher spreading speed, looking at the error bars it can be concluded that the dynamic effect related to such higher speed cause high variability.

## 5. Conclusions

The powder bed spreading process is investigated via discrete element method simulations. The model parameters have been taken from the literature or estimated through a calibration procedure. The DEM model is used to perform a simulation of the spreading process with a realistic setup, including both accumulation and deposition zone. The spreading mechanism is investigated for two different speeds of the blade, corresponding to a quasi-static and an inertial regime. The main results related to the analysis of the state of the powder can be summarized as follows:

- A segregation phenomenon can be observed during both the accumulation and the deposition stage, where the particles with a bigger radius are caught up in the bulk of the powder heap while the smaller particles sink into the powder reservoir and into the powder bed leading to and alteration of the original particle size distribution. Such phenomenon is likely to be more pronounced for the subsequent layers or in case of reusing of the powders.
- For a higher spreading speed, a lower amount of the total powder in the powder reservoir after the spreading process can be observed, further suggesting the necessity to vary the total quantity of powder to be spread.
- A strong variation of the effective layer thickness with a lack of powder in the initial zone of the bed and an accumulation of powder in the terminal zone can be observed for high spreading speed.
- The initial and the terminal zones of the bed are affected in different ways by the interaction with a solid part (the powder bed chamber in this case), thus leading to an alteration of the bed characteristics.
- The described spreading mechanisms are prone to cause further particles segregation during the deposition stage. This appears to be more evident when the speed of the blade is low since, for higher velocities, these segregation phenomena are overwhelmed by the dynamic effects.

The results reported in this work highlight specific mechanisms involved during the blade motion that may be detrimental for the final state of the powder bed. We believe that the present analysis can help to properly set the parameters of the spreading process and design novel printing machines to improve the quality of the spread powder and of the resulting final material.

## Figures and Tables

**Table 1:** Characteristic dimensions of the computational domain.

Dimension	Symbol	Value [μm]
Domain size in *x*-direction	$L_{x}$	3250
Domain size in *y*-direction	$L_{y}$	175
Domain size in *z*-direction	$L_{z}$	1125
Length of accumulation zone	$L_{\mathrm{acc}}$	1250
Length of deposition zone	$L_{\mathrm{dep}}$	1250
Space in between zones	$L_{\mathrm{in}}$	150
Blade width	$W_{\mathrm{blade}}$	250
Layer thickness	$H_{\mathrm{layer}}$	100

**Table 2:** Material properties used in the simulation.

Parameter	Symbol	Value
Density [$kg/m^{3}$]	ρ	9187
Young’s modulus [GPa]	*E*	0.2
Poisson’s ratio	ν	0.3
Coefficient of restitution	*e*	0.7
Coefficient of rolling friction	$\mu_{r}$	0.15

## Data Availability

The data are reported in the article.
